# Activating NRF2E79Q mutation alters the differentiation of human non-small cell lung cancer

**DOI:** 10.21203/rs.3.rs-6606334/v1

**Published:** 2025-05-15

**Authors:** Samera Hamad, Hansa Joshi, T Hess, Stuart Jefferys, Zena Saleh, Rani Sellers, Gord Zhu, Travis Shrank, Rayvon Moore, David Corcoran, Jeremy Simon, Francis Spitz, David Shersher, Michael Major, Bernard Weissman

**Affiliations:** Cooper Medical School of Rowan University; Dana-Farber Cancer Institute; Washington University in St.Louis

**Keywords:** KEAP1/NRF2, lung tumors, cellular transformation, squamous cell carcinoma, neuroendocrine differentiation

## Abstract

The NRF2 signaling pathway promotes tumor initiation, progression and resistance to chemotherapy, radiation therapy and immune checkpoint inhibitors. The mechanisms underlying the biology of NRF2-active tumors are varied, and include altered cellular metabolism, a reductive shift in redox state, and immunosuppression. Here we determined the molecular and phenotypic impact of NRF2 activation on two human non-small cell lung cancer (NSCLC) cell models. Inducible expression of NRF2^E79Q^, a common activating NRF2 mutation, in H358 lung adenocarcinoma (LUAD) cells altered cellular morphology and increased xenograft tumor growth in mice but not in 2D cell culture. In contrast, NRF2^E79Q^ expression in H596 lung adeno-squamous cell carcinoma altered cellular morphology, increased neuroendocrine marker gene expression, but did not impact tumor growth in 2D or in xenografts. Gene expression profiling revealed shared and unique NRF2 transcriptional programs between these models, some of which were shared in primary lung tumors. Collectively, our findings reveal context-dependent effects of NRF2 activation on the growth and differentiation-state of two human NSCLC models, supporting a role for NRF2 activation in altering the differentiation of human NSCLC during tumor progression.

## Introduction

Lung cancer remains the leading cause of mortality worldwide, with an estimated 127,070 deaths in the United States in 2024 [1]. Non-small cell lung cancer (NSCLC) accounts for about 85% of lung cancers: 45% are lung adenocarcinomas (LUAD), 25% are squamous cell carcinoma (LUSC), and 10% are large cell carcinoma (LCC) [2]. Although outcomes for patients with NSCLC have improved over the past 20 years with enhanced radiation protocols and targeted therapies such as immune checkpoint inhibitors (ICIs) and EGFR inhibitors, tumors often develop resistance to these agents [3, 4]

Activating mutations of nuclear factor erythroid 2-related factor-2 (NFE2L2) or NRF2, a transcription factor involved in response to oxidative and electrophilic stress, are found in about 15% of LUSC and 3% of LUAD [5–7]. Beyond its roles in tumor initiation and progression, NRF2 activation is strongly associated with resistance to chemotherapy, radiation and immunotherapy [8–10]. There are numerous reported mechanisms by which NRF2 drives therapeutic resistance, including a reductive shift that abates oxidative damage, direct enhancement of the DNA damage response and suppression of tumor infiltrating lymphocytes. Recently, histological and transcriptional plasticity has been reported to promote therapeutic resistance in numerous cancer types [11–13]. *Therefore, we hypothesized that NRF2 induces the histological transformation in NSCLC*. Our results suggest a role for NRF2 activation in the alterations of differentiation in NSCLC. Results of this study show the histological, transcriptional and growth effects of NRF2 activation in in vitro and in vivo models of the human H358 and H596 NSCLC cell lines. Our data show that NRF2 activation impacts, in a context-dependent fashion, the differentiation state and growth of these cell models.

NRF2 famously induces the expression of genes that protect cells against oxidative and electrophilic stress [14] Early mouse studies showed NRF2 deficiency increased susceptibility to cancer [15, 16]. later GEMM studies and analyses of human tumors implicated NRF2 and its downstream targets in cancer initiation and progression [17–20]. NRF2 is controlled by its negative regulator KEAP1 (Kelch-like ECH-associated Protein 1); KEAP1 forms a complex with NRF2 and CUL3 to prevent the nuclear accumulation and activation of NRF2 by promoting its proteasomal degradation [21]. The Cancer Genome Atlas consortium and other sequencing projects have found that the KEAP1-NRF2 degradation pathway is frequently altered in cancer, by either gain-of-function mutations in NRF2 or loss-of-function mutations in KEAP1 or CUL3 [22, 23]. Most NRF2 mutations localize to hotspots in the DLG and ETGE motifs [24], which impair KEAP1 binding, ubiquitylation and/or subsequent proteasomal degradation [14], yielding constitutively-active NRF2-dependent transcription [25].

To begin to understand the effects of NRF2^E79Q^ expression on the growth and morphology of NSCLC cell lines, we generated H596 LUSC and H358 LUAD cell lines carrying DOX-inducible NRF2^E79Q^. Cell lines were assessed for changes in morphological phenotype, growth and gene expression after NRF2^E79Q^ expression in vitro and in vivo. Activation of the NRF2 signaling by the NRF2^E79Q^ mutation did not impact 2-dimensional (2D) cell growth but did induce morphological changes in both cell lines. In xenograft experiments, NRF2^E79Q^ expression promoted the growth of the H358 LUAD cell line; but H596 tumor growth was unaffected. Immunohistochemistry (IHC) analyses revealed that NRF2^E79Q^ expression altered cellular differentiation, as determined by IHC labeling of p63, NKX2–1 and synaptophysin (SYP). Gene expression signatures enriched after NRF2^E79Q^ activation in the two NSCLC cell lines significantly overlapped with those identified in NRF2-active primary NSCLC tumors. Interestingly and in contrast to H358 cells, the polarity of gene ontology enrichments in the H596 adeno-squamous cell line were sometimes opposite from NRF2-active primary tumors. Thus, the effects of NRF2 activation on gene transcription, differentiation and growth appear to be context-dependent.

## Results

### Activation of NRF2 ^E79Q^ expression changes the morphology of human NSCLC cell lines in vitro.

To investigate if persistent activation of the NRF2^E79Q^ mutation changes the growth and/or differentiation of human NSCLC, we generated stable cell lines harboring a doxycycline (DOX)-inducible expression vector of mutant-NRF2^E79Q^ using pInducer20 (pIND20) system [26]. Because previous studies have shown that DOX exposure can alter cell line growth, morphology, and gene expression [27], we confirmed that DOX (1mg/ml) did not change the protein levels of NRF2 or its downstream targets after 48 hours or the growth after 7 days of the parental cell lines (Supplementary Fig. 1a-c). Further, we did not observe changes in cell morphology of the parental H596 and H358 cell lines after DOX treatment (data not shown). Therefore, we confirmed that changes observed after DOX-induced expression of NRF2^E79Q^ did not result from the DOX treatment.

**Figure 1:**
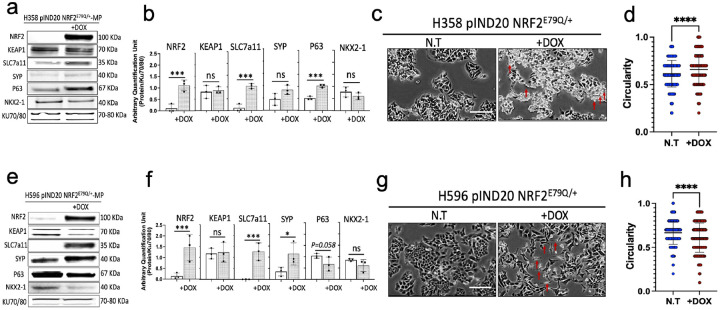
Changes in differentiation of human NSCLC cell lines after NRF2^E79Q^ induction in vitro. **a** Immunoblotting showing induction of NRF2^E79Q^ mutation with its downstream targets and differentiation markers in H358 pIND20 NRF2^E79Q/+^-MP, with **b** Protein quantification; **c** Changes in cell morphology observed 96 hours after DOX treatment (1mg/ml) to activate NRF2^E79Q^ expression in H358 pIND20 NRF2^E79Q/+^-MP, **d** quantification of H358 pIND20 NRF2^E79Q/+^-MP cell circularity −/+ DOX treatment, **e** Immunoblotting showing induction of NRF2^E79Q^ mutation with its downstream targets and differentiation markers, with **f** protein quantification, **g** Changes in cell morphology observed 96 hours after DOX treatment (1mg/ml) to activate NRF2^E79Q^ mutation in H596 pIND20 NRF2^E79Q/+^-MP, and **h** quantification of H358 pIND20 NRF2^E79Q/+^-MP cell circularity −/+ DOX treatment. Protein was quantified by image J; KU70/80 served as loading control. Error bars in **b** and **e** represent 3 biological replicates. Scale bar=200 μm. Cell circularity in **d** and **h** was measured using ImageJ, the unit is a dimensionless number calculated as 4π * (Area / Perimeter^2), with a value of 1 representing a perfect circle. Red arrows show changes in circularity/ morphology, *p<0.05, **p<0.01, ***p<0.001

We also confirmed the activation of NRF2 signaling in both cell lines after DOX treatment by western blotting. Both cell lines showed increased protein levels of NRF2 and representative key target proteins (Fig. 1a–b, e–f and Supplementary Fig. 2a-d). Interestingly, immunoblotting showed that treatment with CDDO (Bardoxolone methyl, 100nM [28]), a non-specific activator of NRF2, induced expression of HMOX1, a common NRF2 target gene, in the H596 cell line while NRF2^E79Q^ induction did not (Supplementary Fig. 2b) [29]. On the other hand, expression of another common target gene of NRF2, the cysteine/glutamate antiporter SLC7A11, was induced by NRF2^E79Q^ induction, but not CDDO in the H358 cell line (Supplementary Fig. 2d). These findings suggest wild-type NRF2 and the mutant NRF2^E79Q^ may regulate common downstream targets as well as unique ones, consistent with the large number of genes altered by CDDO treatment compared to expression of the NRF2^E79Q^ [30].

We next tested whether activation of NRF2 signaling impacted cellular morphology. Both NSCLC cell lines with pIND20-NRF2^E79Q^ showed morphology changes after 48 hours of DOX exposure (Supplementary Fig. 2a,c). To better understand the effects of NRF2^E79Q^ expression on the appearance of both NSCLC cell lines, we extended the exposure of the cell lines to DOX to 96 hours (Fig. 1c,g). Both cell lines showed significant changes in morphology, where H596 cells became more elongated/less circularity, while H358 cells became smaller with more cell circularity (Fig. 1c–d,g–h and Supplementary Fig. 3a,b). These results suggest that activated NRF2 signaling can change the differentiation of human NSCLC cell lines in vitro. Consistent with this notion, we found that NRF2^E79Q^ expression induced a 2–3 fold increase in p63, a marker of squamous cell differentiation (Fig. 1a,b). However, we did not see altered NKX2–1 expression, a marker of LUAD differentiation (Fig. 1a,b). For the H596 cell line, we observed significantly increased expression of the neuroendocrine marker synaptophysin (SYP) and a near-significant decrease in P63 expression (p = 0.058) with no change in NKX2–1 expression (Fig. 1e,f).

### Induction of the NRF2 ^E79Q^ mutation in the H358 LUAD cell line increases their growth in vivo.

**Figure 2:**
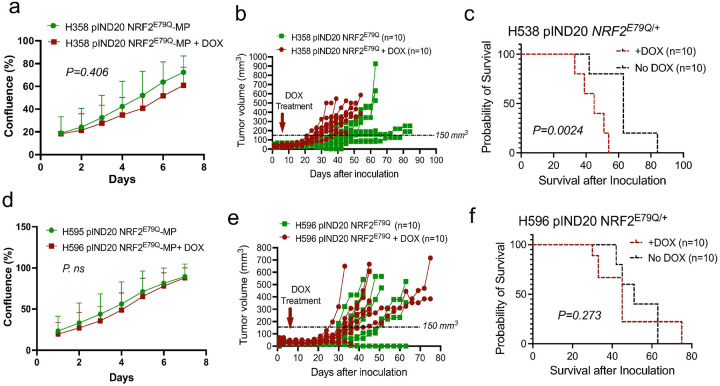
Tumor growth of human NSCLC cell lines in vitro and in vivo ± NRF2^E79Q^ induction. **a** 2D-growth curve of H358 pIND20 NRF2^E79Q/+^-MP, **b** Tumor growth (xenograft) of H358 pIND20 NRF2^E79Q^ ± doxycycline (DOX, 2mg/ml + 1% sucrose in water) after subQ inoculation into nude mice shows significant changes in growth +DOX, **c** survival curve of nude mice after inoculation with H358 pIND20 NRF2^E79Q^ (±DOX) using subQ injection, with worse survival in mice after NRF2 activating mutation, **d** 2D-growth curve of H596 pIND20 NRF2^E79Q/+^-MP ±DOX, and **e** Tumor growth (xenograft) of H596 pIND20 NRF2^E79Q^ ±DOX in mice, shows no significant changes in growth +DOX compared to no DOX treatment. N=10 tumors per group, and **f** survival curve of nude mice after inoculation with H596 pIND20 NRF2^E79Q^ (±DOX) with no significant impact from NRF2^E79Q^ activating mutation on survival, N=10 tumors per group, error bars in **a** and **c** represent 3 technical replicates across 3 biological replicates.

**Figure 3:**
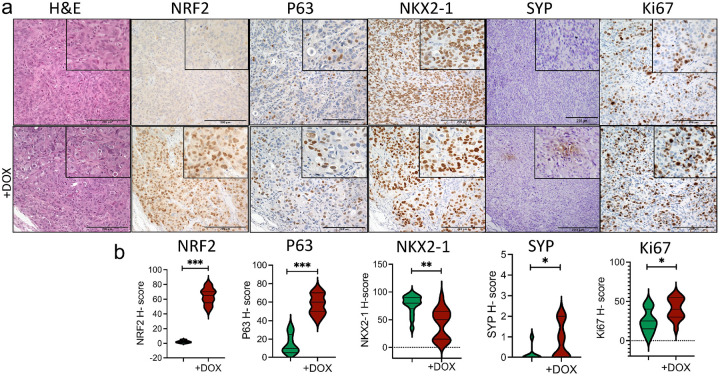
In vivo induction of NRF2^E79Q^ in human lung adenocarcinoma (H358 pIND20 NRF2^E79Q^-MP) cell line. **a** H&E staining and IHC labeling of NRF2, P63 (squamous cell carcinoma marker), NKX2–1 (adenocarcinoma marker), synaptophysin (SYP, neuroendocrine marker), and Ki67 (a proliferative marker). scale bar=200mm, SYP: few areas from H358 pIND20NRF2^E79Q^-MP showing SYP after Doxycycline (DOX) treatment which we did not observe in the control; **b** H-score of markers presented in **a,** DOX was provided to nude mice at 2mg/ml in water + 1% sucrose. *p<0.05, **p<0.01, ***p<0.001.

Across many NSCLC models, NRF2 has a limited impact on 2D growth but a pronounced effect on 3D cell growth. For instance, studies have shown that increased expression of wild-type NRF2 through loss of KEAP1 function increased the growth of LUAD cells significantly in 3D culture [31]. In this study, we tested the effect of activating NRF2^E79Q^ mutation on the growth of two NSCLC cell lines. The expression of the NRF2^E79Q^ mutation had no effect on the growth of the H358 cells in 2D culture (Fig. 2a). We also assessed the effects of NRF2^E79Q^ expression on the in vivo growth of the H358 cell line in subcutaneous xenografts in nude mice. When tumors reached ~ 150 mm^3^, DOX (at 2 mg/ml + 1% sucrose in water) was provided to one-half of the mice to induce NRF2^E79Q^ expression. We found that the induction of NRF2^E79Q^ significantly increased the growth of the H358 cell line in nude mice as evidenced their decreased survival as compared to the untreated mice (Fig. 2b,c). We characterized these tumors for expression of NRF2 by immunohistochemistry (IHC) and observed a strong induction of nuclear NRF2 expression (Fig. 3a,b). The proliferative marker (Ki67) was significantly higher in the NRF2^E79Q^-H358 tumors compared to controls, consistent with an increased growth rate leading to their poorer survival (Fig. 3a,b and Fig. 2b). We also saw increased expression of p63, similar to that observed in cell culture, along with a decrease in NKX2.1 expression. While these results suggest that NRF2^E79Q^ expression can induce the transformation from LUAD to LUSC, we did not observe a change in LUAD or LUSC gene signatures by RNA-seq (data not shown).

**Figure 4:**
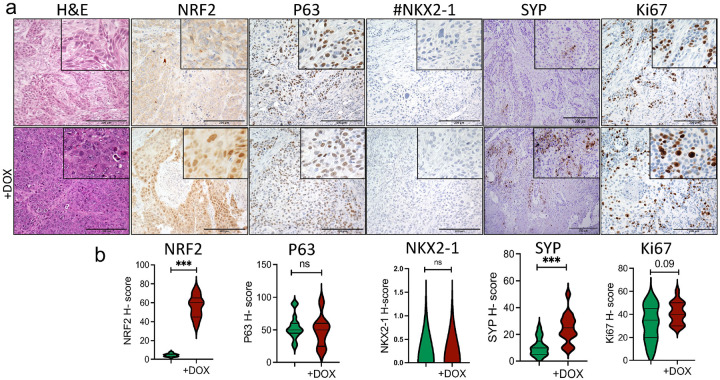
In vivo induction of NRF2^E79Q^ in human adeno-squamous cell carcinoma (H596 pIND20 NRF2^E79Q^-MP) cell line. **a** H&E staining and IHC labeling of NRF2, P63 (squamous cell carcinoma marker), NKX2–1 (adenocarcinoma marker), synaptophysin (SYP, neuroendocrine marker), and Ki67 (a proliferative marker). scale bar=200mm, ^#^both H596 pIND20 NRF2^E79Q^-MP with and without Doxycycline (DOX) treatment were negative for NKX2–1; **b** H-score of markers presented in **a**, DOX was provided to nude mice at 2mg/ml in water + 1% sucrose. ***p<0.001.

In contrast, the expression of NRF2^E79Q^ did not impact the proliferation of H596 cells in vitro (Fig. 2d), tumor growth in vivo (Fig. 2e), or survival (Fig. 2e,f). While H596 cells expressing NRF2^E79Q^ did not have increased p63 expression or induce NKX2.1 expression (Fig. 4a,b), they did show synaptophysin (SYP) expression, a neuroendocrine marker, in vitro and in vivo (Figs. 1e,f & 4a,b).

### GSE pathway analysis in NSCLC primary tumors and cell lines implicates cellular context in the regulation of NRF2 signaling

To better characterize effects of NRF2^E79Q^ expression on the molecular profiles of the NSCLC cell lines, we performed RNA-sequencing (RNA-seq) on at least five H358 and H596 cell line, −/+ NRF2^E79Q^, tumors (Supplementary Fig. 4). We first confirmed the induction of NRF2 signaling by gene-set enrichment analysis (GSEA) using a custom NRF2 gene-set signature constructed from previous reports (Supplementary Fig. 5a,b) [32, 33]. To complement these data, we also identified primary human LUSC and LUAD tumors from the TCGA with *NRF2, KEAP1*, and *CUL3* mutations using DNA-seq data that was manually downloaded from the Genomic Data Common web interface [34] (Supplementary Table 1). We then analyzed the corresponding RNA-seq data for these tumors using GSEA to compare tumors with mutations in genes that activate NRF2 signaling (*NRF2, KEAP1,* or *CUL3*) to those tumors with no mutations in these genes. Again, the NRF2 signature was enriched in both human LUAD and LUSC primary tumors with mutations in genes that activate NRF2 signaling versus tumors lacking these mutations (Supplementary Fig. 5c,d).

**Figure 5:**
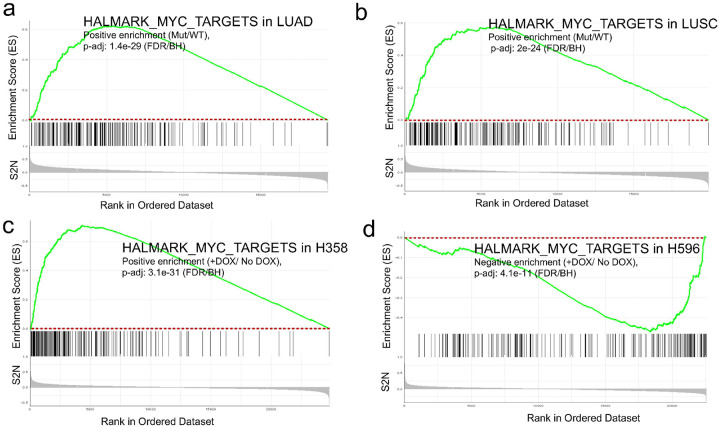
Enrichment score of MYC pathway signature in primary human tumors and human NSCLC cell lines used in this study. **a** Hallmark MYC signaling pathway is significantly enriched in mutant (Mut)-NRF2 human lung adenocarcinoma (LUAD) tumors, and **b** Mut-NRF2 human lung squamous cell carcinoma (LUSC) tumors compared to wild type (WT) tumors; **c** MYC signaling pathway is also significantly enriched after NRF2^E79Q^ activation, by doxycycline (DOX, 1mg/ml) treatment, in human lung adenocarcinoma (H358) cell line tumors, but not in **d** human lung adeno-squamous cell carcinoma (H596) cell line tumors, where the MYC signaling is enriched in low-NRF2 tumors from H596 cell lines.

**Figure 6:**
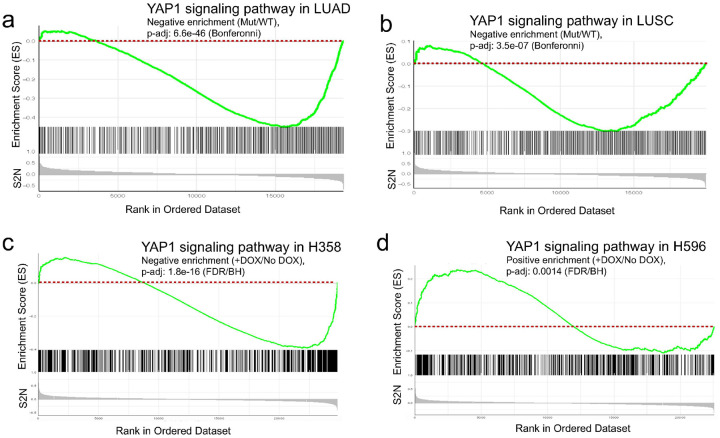
Enrichment score of YAP1 signaling pathway in primary human tumors and human NSCLC cell lines used in this study. **a** YAP1 signaling pathway is not enriched in the mutant (Mut)-NRF2 human lung adenocarcinoma (LUAD) tumors, or **b** Mut-NRF2 human lung squamous cell carcinoma (LUSC) tumors compared to wild type (WT) tumors; **c** YAP1signaling pathway is also not enriched after NRF2^E79Q^ activation, by doxycycline (DOX, 1mg/ml) treatment, in human lung adenocarcinoma (H358) cell line tumors, but is significantly enriched in **d** human lung adeno-squamous cell carcinoma (H596) cell line tumors after NRF2^E79Q^ activation.

To better understand the pathways altered by activated NRF2 signaling in primary human LUAD and LUSC tumors and in H358 and H596 cell line xenografts, we applied GSEA to the RNA-seq data using Curated, GeneOntology, and Hallmark genesets (Supplementary Tables 2&3). Results demonstrated that many pathways showing significant changes in enrichment between mutant NRF2 and WT NRF2 LUAD and LUSC primary aligned with our H358 and H596 xenografts with and without NRF2^E79Q^ activation while others were discordant (Table 1, Supplementary Table 4). Surprisingly, while the LUAD and LUSC tumors and the H358 xenografts had similar directions of enrichment across these gene signatures, H596 xenografts demonstrated the opposite direction of enrichment of some of these gene signatures (Table 1). For example, two oncogenic pathways often altered in LUAD and LUSC, MYC and YAP1, had positive and negative enrichment, respectively, in the H358 xenografts, and LUAD and LUSC primary tumors (Figs. 5a–c, and 6a–c). In contrast, the H596 xenografts with NRF2^E79Q^ expression had negative enrichment of the MYC pathway and positive enrichment of the YAP1 pathway (Figs. 5d and 6d).

Because of the induction of the neuro-endocrine marker, SYP, in the H596 after NRF2 activation, we also looked at a NE differentiation signature in our RNA-seq data. While we observed a weak but statistically-significant enrichment of this signature in the H596 cell lines, we did not observe enrichment in the LUSC primary tumors (Supplementary Fig. 6).

## Discussion

Our recent GEMM studies implicated expression of the NRF2^E79Q^ mutation, one of the most common mutations found in human tumors [35], in the initiation and/or progression of different tumors, including oral SCC, cutaneous SCC, and SCLC in mice [19, 20, 30]. Here, we report the effects of NRF2^E79Q^ expression on the growth and differentiation of two human NSCLC cell lines. We also compared gene expression between NRF2-active and NRF2-wild-type NSCLCs from the TCGA. Our results demonstrate that expression of the activated NRF2^E79Q^ increased the growth rate of H358 cells in vivo but had no effect on the growth of the H596 cell line. These findings expand the range of effects of NRF2 activation in human NSCLC. We observed similar effects on downstream targets and signaling pathways after NRF2^E79Q^ expression in the NSCLC cell lines and in the NRF2-active primary LUSCs and LUADs.

Previous studies have suggested that the population of cells displaying squamous cell differentiation in lung adeno-squamous cell carcinomas, representing 0.4–4% of NSCLC, may represent a major pathway to development of pure LUSC [36]. Clinical studies have also suggested that resistance to chemotherapy associated with transformation from LUAD to LUSC may be driven by the emergence of preexisting LUSC cells within an adeno-squamous cell carcinoma [11, 36]. Our western blotting, IHC, and RNA-seq results suggest that activated NRF2 signaling could induce a neuroendocrine differentiation program in the H596 (lung adeno-squamous carcinoma) cell line (Figs. 1e,f, 4a,b and Supplementary Fig. 6d). In the H358 (LUAD) cell line, we could not confirm an effect on cellular differentiation by NRF2^E79Q^ expression, despite increased TRP63 expression in culture (Fig. 1a,b), and in tumors (Fig. 3a,b). Of importance, IHC staining remains the gold standard for tumor differentiation and diagnosis [37]. Therefore, IHC-labeling of lung tumors with markers specific for NRF2, along with NE, SCC and LUAD markers is recommended before and after treatment to monitor for transformation to more aggressive tumors (i.e. SCLC and LUSC).

Data analysis of primary human LUAD and LUSC tumors from the TCGA showed that tumors with active NRF2 signaling showed significant enrichment of classical oncogenic pathways such as MYC and YAP1 [38, 39] (Table 1, Figs. 5a,b, 6a,b). Remarkably multiple signaling and prognostic gene signatures, including MYC and YAP1, were also significantly enriched after NRF2^E79Q^ induction in the H358 and H596 cell lines (Table 1, Figs. 5a–d, 6a–d). However, while NRF2^E79Q^ induction in the H358 cell line caused the same direction of enrichment as those observed in the primary tumors, most gene signatures in H596 after NRF2^E79Q^ induction showed the opposite direction (Table 1, Figs. 5a–d, 6a–d). The differences in MYC pathway enrichment might explain the absence of growth effects by activation of NRF2 signaling in the H596 cell lines and xenografts (Fig. 2d,e,f). The increased enrichment of YAP signaling in the H596 cell line after NRF2 activation suggests that crosstalk previously observed between the NRF2 and YAP signaling pathways acts in an additive fashion (Fig. 6d, and Supplementary Fig. 5b) [40]. In contrast, the two pathways may function in an antagonistic manner in the H358 cell line and the LUAD and LUSC primary tumors (Fig. 6a–c, and Supplementary Fig. 5a,c,d).

Previous mouse studies in our lab showed activation of Nrf2^E79Q^ supports SCLC development [20]. Here, NRF2^E79Q^-active H596 tumors with RB/P53 mutations (that drive human SCLC) showed trans-differentiation towards SCLC, most likely SCLC-Y type with high YAP expression and low MYC signaling [40]. In contrast, NRF2^E79Q^-active H358, LUAD tumors with *P53/KRAS* mutations, showed histological transformation to LUSC, by IHC and WB. These tumors had low YAP and high MYC signaling, which might explain the high growth rate in these tumors [41, 42]. These findings could be explained by the negative regulatory relationship between YAP and MYC [43]. Also, *NRF2*^E79Q^ activating mutation may interact with other mutations such as CTNNB1 mutation in H358, and the PIK3CA mutation in H596 which could impact MYC and YAP1 signaling and thus impacts the trans-differentiation in NSCLC directly or indirectly [41, 42, 44–46].

Several possibilities could account for the differential responses of the H596 cell line to activation of NRF2 signaling compared to the changes found after its activation in the H358 cell line and the primary tumors. As an adeno-squamous cell line, H596 contains a mixed population of cell types as shown in Fig. 4a. In addition, the different driver mutations found in the H358 and H596 cell lines may alter the impact of NRF2 signaling. Consistent with the last mechanism, the H358 cell line possesses mutations in common driver genes for LUAD-*KRAS* and *TP53*, while the *H596* cell line carries TP53 and RB1 mutations (Supplementary Fig. 1d), [7, 47, 48].

The effects of the target genes induced by the NRF2 transcription factor remain under explored. The differences in NRF2-driven gene expression in different tumors may prove key markers for the efficacy of targeted therapies. To add to this complexity, we found that two known NRF2 target genes, *HMOX1* and *SLC7a11*, were activated only by wild type NRF2 or by NRF2^E79Q^ induction, respectively (Supplementary Fig. 2b, d). Indeed, Levings et. al., identified a set of genes activated by NRF2 specifically in human tumors [49]. Thus, the targets of the wild type NRF2 protein and its mutant forms may differ, suggesting tumors carrying KEAP1 inactivating mutations versus those with NRF2 activating mutations can show different phenotypes [50].

Our current study has several limitations. We did not use a human LUSC cell line to study the effects of NRF2^E79Q^ induction because common human LUSC cell lines, such as H520 and SK-MES-1, already possess high levels of NRF2 signaling [31]. Therefore, we only observed small increases in the levels of NRF2 protein and its downstream targets after expression of NRF2^E79Q^ (data not shown). Also, we considered all mutations that appeared in the *CUL3* and *KEAP1* genes as activators of constitutive NRF2 signaling when identifying NRF2 active and NRF2 wild-type primary LUAD and LUSC tumors, while many of these mutations have not been formally shown to activate NRF2 signaling in model system. However, the NRF2 active set of LUSC and LUAD tumors did show significant positive enrichment of NRF2 signaling by GSEA (Supplementary Fig. 5c, d).

Taken together, our results reveal the complex effects of the NRF2^E79Q^ mutation on the growth of human LUAD and LUSC cells and on gene expression programs associated with tumor differentiation. Clinical studies have also shown that a switch in NSCLC differentiation to a NE phenotype is associated with emergent treatment resistance to epidermal growth factor (EGFR) and other tyrosine kinase inhibitors (TKIs) [51]. Additionally, several cases with a change in differentiation from LUAD to LUSC have been reported, either spontaneously [52] or subsequent to TKI [53], anaplastic lymphoma kinase (ALK) inhibitor [54], or immunotherapy [55] treatment. Our results suggest a role for NRF2 activation in these alterations of differentiation of NSCLC. It may be that pre-existing NRF2 mutations in some tumors render them more susceptible to phenotype switching, and thus more likely to develop resistance. Alternatively, activating NRF2 mutations may be acquired during treatment with radiation therapy, chemotherapy or targeted therapy. Future studies will investigate the spectrum of NRF2 mutations in NSCLC patient samples using IHC and WGS before and after treatment to understand the timing and their effects on molecular subtypes. Ongoing studies in our lab are investigating the linear tracing of these transformations using in vitro and in vivo (immune deficient mouse) models. We also treat these models with chemotherapy before and after activating NRF2^E79Q^ mutation to better understand how the interaction between NRF2 activation and chemotherapy may impact histological and transcriptional transformation and responses to treatment. Future studies will use scRNA-seq to better understand these changes. Further, targeting active NRF2 signaling, before or during treatment, may provide an important approach to blocking transformation to these more aggressive tumors.

## Materials and Methods

### Cell Lines and Culture

All the cell lines were obtained from ATCC (American Type Culture Collection) and authenticated by Cell Line Authentication and Research Services from Labcorp by detecting short tandem repeat (STR) markers. Cell lines were also tested for mycoplasma every 6 months. The parental cell lines were maintained in Roswell Park Memorial Institute (RPMI-1640) medium (#11875119, Thermo Fisher Gibco) with 10% BenchMark^™^ fetal bovine serum (#100–106, GeminiBio). The two cell lines, H358 and H596 shared TRP53 mutation while H358 had KRAS mutation, and H596 possessed inactivation of RB1 (Supplemental Fig. 1d). The H358 and H596 cell lines transduced with pINDUCER were maintained in RPMI medium with 10% BenchMark^™^ fetal bovine serum and 200 mg/mL of G418 (#10121035, Thermo Fisher Scientific). All cells were incubated at 37°C in a humidified and 5% CO_2_ atmosphere.

#### pINDUCER20

Lentivirus was generated using 293FT cells as previously described [26]. Briefly, pINDUCER20-TRE2-Nfe2l2^E79Q^ were transfected with the packing construct ΔNRF (from Dr. Tal Kafri, University of North Carolina; [56]) and the VSV-G envelope expression plasmid (pMDK64; from Dr. Matthias Kaeser, Salk Institute) into 293FT cells. Lentiviral particles were collected 48 hours later and used for infection of the H596 and H358 cell lines. After overnight incubation, cells were selected in RPMI 1640 medium supplemented with 10% BenchMark^™^ FBS and 200 mg/mL G418 to create doxycycline inducible cell lines. Cells were tested for NRF2^E79Q^ expression after the addition of doxycycline (1mg/mL) by immunoblotting to confirm the expression of NRF2 through E79Q mutation.

#### Assessment of cell circularity

To quantitatively assess cell circularity, ImageJ, an open-source image analysis software, was used. High-resolution microscopy images of H358 pIND20 NRF2^E79Q/+^ and H596 pIND20 NRF2^E79Q/+^ were processed at 96 hours after DOX treatment (1mg/ml) using thresholding techniques to delineate cell boundaries, images with no DOX treatment were used for comparison. The “Analyze Particles” function was employed to measure circularity, defined as 4π×(area/perimeter2) [57], where a value of 1 indicates a perfect circle, and values approaching 0 represent increasingly elongated or irregular shapes [57]. Standardized image processing parameters were applied to ensure consistency across samples, minimizing user bias [57].

### Confluency Assay

To test the effect of doxycycline (DOX, 1mg/ml) on in vitro cell growth of both H358 and H596 parental cell lines, we seeded 5 × 10^4^ cells per well, in 24-well plate. Cells were incubated overnight, with doxycycline (DOX, 1mg/ml) added to DOX-labeled cells at 24 hours. Plates were placed in a IncuCyte S3 instrument located in a humidified incubator at 37°C with 5% CO_2_. Cell growth/confluency was measured for 7 days (Supplementary Fig. 1c). Cell growth of H358 pIND20 NRF2^E79Q/+^-MP and H596 pIND20 NRF2^E79Q/+^-MP with and without NRF2^E79Q^ activation was also performed using the same protocol. Three technical replicates across three biological replicates were performed for all experiments (Fig. 2a,d).

#### Protein Extraction and Immunoblotting

Protein was extracted using urea protein extraction method. Harvested cell pellets were resuspended in 1mL of cold 1x phosphate-buffered saline (PBS) and transferred to Eppendorf tubes. This was centrifuged at 10,000 RPM for 5 min at 4C and the supernatant was aspirated. Repeat PBS wash was performed. Urea Protein extraction buffer was added to 2–3x the cell pellet size. The buffer consists of final concentrations of 8.0M Urea, 0.1M sodium phosphate, 10mM Tris pH 8.0, βME 0.1%. After centrifuging, the supernatant, which contains the protein, was preserved. Protein concentrations were determined by Bradford protein quantification (Pierce).

Proteins were separated on Invitrogen NuPAGE 4 to 12% Bis-Tris Gel (#NP0321BOX, Thermo Fisher Scientific) in NuPAGE MOPS SDS Running Buffer (#NP0001, Thermo Fisher Scientific) at 120V for 1.5 hours. Proteins were transferred to a nitrocellulose membrane (#1620115, Bio-Rad) using NuPAGE transfer buffer (#NP0006, Thermo Fisher Scientific) at 24V for 1 hour. Ponceau S staining was used to determine efficacy of transfer. After blocking in 5% nonfat dry milk in 1X PBS for 1 hour, the membrane was washed using 1X PBS with Tween 20 (PBST) (3 × 10 minutes), cut, and each membrane section incubated overnight at 4°C in the primary antibodies (Supplementary Table 5). After primary incubation, membranes were washed in 1x PBST (3 × 10 minutes) and the membranes were incubated in ECL secondary antibody for 1.5 hours at room temperature. Proteins were visualized with Clarity Western ECL Substrate (Bio-Rad) and imaged using iBright Imaging System (Thermo Fisher Scientific). We used positive and negative controls in each run. We also used 3 biological replicates of each cell line.

#### In vivo Studies

To assess the effects of the NRF2^E79Q^ mutant on the growth and differentiation of the H596 and H358 cell lines in vivo, we inoculated the H596 pIND20 NRF2^E79Q^-MP or H358 pIND20 NRF2^E79Q^-MP into both flanks of N = 10 female nude mice (purchased from the UNC-LCCC Animal Studies Core) at 6–8 weeks of age [58]. Female recipient mice were used as they are more stable than male in terms of behavior [59]. We inoculated 5×10^6^ cells subcutaneously in two sites per mouse, as previously described [58]. When tumors reached a size of ~ 150 mm^3^, 1/2 of the mice were provided DOX in water (2 mg/ml + 1% Sucrose) to activate the NRF2 signaling in the tumor cells. Mice were sacrificed upon signs of distress (i.e., labored breathing and/or weight loss or other body conditions such as fur ruffling, difficulty in walking and hunched posture) or 8 weeks after DOX-treatment, and tumors were collected and fixed in 10% neutral buffered formalin. The UNC-LCCC Animal Studies Core performed the monitoring, weighing, and isolation of tumors of the mice. All mouse work was performed under an approved IACUC protocol at the University of North Carolina Chapel Hill (# 19–242.0).

### Tissue processing, Hematoxylin & Eosin (H&E), and immunohistochemistry (IHC) labeling

Harvested tissues were fixed via submersion in 10% neutral buffered formalin at room temperature. Tissues were sent to the UNC-LCCC Pathology Services Core (formerly known as the Animal Histopathology Core) for routine processing to paraffin wax, sectioning, and staining. Paraffin sections of 5 μm were stained using hematoxylin and eosin (H&E). Additional 5 μm sections were prepared for immunohistochemistry (IHC) labeling as previously described [60]. Briefly, for H&E staining, the paraffin embedded tissue was sectioned (5 μm thickness), de-paraffinized, and rehydrated using xylene, ethanol and dH_2_O. For hematoxylin staining, slides were rinsed in hematoxylin, water, then ethanol; slides were then eosin stained, then rinsed in ethanol and xylene. IHC labeling was performed on the Ventana’s Discovery Ultra Automated IHC platform using the following primary antibodies: NRF2, ab137550, Abcam (1:500); TTF1/NKX2–1, ab133737 Abcam (1:1000); TRP63, 12143–1, Protein Tech (1:25); TRP53, VP-P956, Vector Laboratories, Inc (1:500), and synaptophysin (SYP), sigma (1:100). Discovery OmniMap anti Rabbit HRP (760–4311, ready to use) was incubated for 30 minutes at room temperature, specimens were treated with 3,3’-diaminobenzidine (DAB) and Hematoxylin II for 12 minutes, followed by Bluing Reagent for 4 minutes.

Samples were microscopically evaluated by a board-certified veterinary pathologist (RSS) in a masked fashion to assess morphology and IHC staining. IHC scoring was based on (1) the estimated precent of positively labeled cells within the tumor section; and (2) the overall intensity of the staining (Supplementary Table 6).

### RNA-sequencing

Total RNA was extracted from harvested from flash frozen H596 and H358 tumors, ± DOX, as previously described [61] using TRIZOL (Invitrogen, 15596026) following the manufacturer’s protocol. Samples were sent to Novogene Corporation Inc. (Novogene, Sacramento, CA) for sequencing where the Agilent 2100 Bioanalyzer (Agilent Santa Clara, CA) was used to test for RNA samples integrity and purity. Subsequently, 250–300 base pair (bp) insert cDNA libraries was generated and sequenced.

### RNA-data analysis

Raw FASTQ files were aligned to the GRCh38v102 version of the human genome and transcriptome using the STAR short read aligner. Quantification of gene abundance for each sample was done using Salmon. Genes were filtered to keep those with 10 or more reads in at least one samples. Normalization and differential gene expression (DE) was performed using DESeq2 v1.36.0. Bioconductor v3.15 [58], and R v4.2.1. Gene set enrichment results [62] were generated using the fgsea v1.22.0 Bioconductor package with gene sets ‘Hallmark’, ‘Curated Pathways’, and ‘Gene Ontologies’ from the MSigDB. Reported p-values for pathways in these gene sets were adjusted for false discovery rate (FDR), separately for each DE data set.

Heatmaps were generated using normalized expression data filtered to keep only significantly differentially expressed genes at an FDR of < 0.05, log transformed, split by cell-line of origin, z-scaled, and then visualized using the Complex-Heatmap package v2.12.1 with spearman correlation-based sample and gene hierarchical clustering. DOX vs NO-DOX gene-set enrichment results were generated by fgsea v1.22.0 using the DESeq2 Wald statistic from the DE analysis to rank differential enrichment for selected gene sets in each of the four data sets. Running score gene rank plots were generated using a custom R script and annotated with p-values FDR corrected within each data set.

#### Human NSCLC data analysis.

The Genomic Data Common web interface [34], was used to download RNA-seq expression data for the 539 available TCGA-LUAD [6], and 502 TCGA-LUSC [7] samples. Mutation data for the same projects were downloaded from cBioPortal [5]. The expression data was filtered using R v4.3.1 and Bioconductor v3.18 to the unstranded count data from protein-coding genes with 10 or more counts in at least one gene. Only count data from the most recent aliquot for which NRF2 pathway mutation data were also available was retained. Samples were split into mutant “mut” KEAP1-NRF2 pathway where KEAP1, NRF2 or CUL3 was found to be mutant, and wild type “wt” categories, where no mutation was observed in any of the KEAP1-NRF2 component. Differential gene expression (DGE) analysis was carried out within each cancer type comparing these two groups using DESeq2 v1.42.0. Gene set enrichment analysis was performed on four specified genes sets using fgsea v1.28.0, with ranks defined by the Wald test statistic from the DGE analysis. Running score gene rank plots were generated using a custom R script and annotated with p-values Bonferroni corrected within each cancer type.

### Statistical analysis

We used GraphPad Prism 8 software (GraphPad Software, San Diego, CA, USA) to present the data. Fishers Exact Test was used to identify the differences between groups. A P value less than 0.05 (typically ≤ 0.05) was considered statistically significant.
